# Detection of *Rickettsia* spp. in *Rhipicephalus sanguineus* (*sensu lato*) collected from free-roaming dogs in Coahuila state, northern Mexico

**DOI:** 10.1186/s13071-019-3377-z

**Published:** 2019-03-26

**Authors:** Aldo I. Ortega-Morales, Erika Nava-Reyna, Verónica Ávila-Rodríguez, Vicente H. González-Álvarez, Antonio Castillo-Martínez, Quetzaly K. Siller-Rodríguez, Alejandro Cabezas-Cruz, Filipe Dantas-Torres, Consuelo Almazán

**Affiliations:** 1 Departamento de Parasitología, Universidad Autónoma Agraria Antonio Narro, Unidad Laguna, Coahuila, Mexico; 2 Instituto Nacional de Investigaciones Forestales, Agrícolas y Pecuarias, Durango, Mexico; 3 Laboratorio de Entomología, Facultad de Ciencias Biológicas, Universidad Juárez del Estado de Durango, Durango, Mexico; 4 Ingeniería en Innovación Agrícola Sustentable, Instituto Tecnológico de Santa María de El Oro, Santa María de El Oro, Durango, Mexico; 5 UMR BIPAR, INRA, Ecole Nationale Vétérinaire d’Alfort, ANSES, Université Paris-Est, 94700 Maisons-Alfort, France; 6 Department of Immunology, Aggeu Magalhaes Institute, Oswaldo Cruz Foundation (Fiocruz), Recife, Brazil

**Keywords:** *R. rickettsii*, *R. rhipicephali*, *R. sanguineus* (*s.l.*), Coahuila, Mexico

## Abstract

**Background:**

The aim of this study was to detect and molecularly identify *Rickettsia* spp. in *Rhipicephalus sanguineus* (*sensu lato*) collected from free-roaming dogs in 30 communities from five municipalities in the south of Coahuila State, northern Mexico, where Rocky Mountain spotted fever is endemic.

**Methods:**

In total, 60 dogs from each municipality were examined for engorged ticks. DNA was isolated from tick pools and conventional PCR assays targeting the 23S-5S ribosomal RNA intergenic spacer and outer membrane protein (*ompA*) gene of *Rickettsia* spp. were performed.

**Results:**

All ticks (*n* = 1238) were morphologically identified as *R. sanguineus* (*s.l.*). Six pools (each with six engorged females) from four municipalities were positive to *Rickettsia* spp. DNA sequencing and phylogenetic analyses confirmed the presence of *R. rickettsii* and *R. rhipicephali* in *R. sanguineus* (*s.l.*) in these ticks.

**Conclusions:**

This study confirms the presence of *R. rickettsii* and *R. rhipicephali* in *R. sanguineus* (*s.l.*) from stray dogs in the south of Coahuila. This suggests that stray dogs may play a role in the inter-municipal dissemination of infected ticks in this region. Further research is required to assess whether ticks from stray dogs could serve as good indicators for the molecular xenomonitoring of *R. rickettsii* in this region. Considering that *R. sanguineus* (*s.l.*) is a proven vector of *R. rickettsii* in Mexico, increased awareness regarding permanent tick control in dogs is warranted.

## Background

*Rickettsia rickettsii* is the causative agent of Rocky Mountain spotted fever (RMSF), a tick-borne disease with increasing incidence rate in North, Central and South American countries, including Mexico [1–3]. RMSF is the most common rickettsial disease affecting human beings in Mexico, with devastating outbreaks detected in several communities in the states of Sinaloa, Sonora, Durango and Coahuila [3]. For instance, Coahuila reported an estimated incidence rate of RMSF of three cases per 100,000 inhabitants in 2014, which corresponds to the highest incidence rate reported in Mexico during that year [4]. It is worth mentioning that the possibility of underreporting is high, especially because RMSF cases are often misdiagnosed with other diseases that cause flu-like symptoms, including dengue fever [5].

Recent epidemics of RMSF in south-western USA and northern Mexico have been associated to massive environmental infestations by brown dog ticks, *Rhipicephalus sanguineus* (*sensu lato*) [1, 3, 6]. Brown dog ticks are recognized as important vectors of several pathogenic organisms in south-western USA and northern Mexico, including *R. rickettsii* [1, 3, 6, 7], and are the most frequent ectoparasites of dogs [1, 3, 6–8]. For instance, *R. sanguineus* (*s.l.*) is commonly found infesting both privately-owned and stray dogs in urban and rural areas in Mexicali, the capital of Baja California state, northern Mexico [8], where an epidemic of RMSF was documented in 2008, and has since affected approximately 4000 people as of 2018 [6].

In recent outbreaks of RMSF associated with brown dog ticks (e.g. in eastern Arizona, south-western USA [1]), most human cases were diagnosed in communities with large numbers of free-roaming dogs and brown dog ticks. Incidentally, free-roaming dogs are common in northern Mexico and could play a role in disseminating *R. rickettsii*-infected brown dog ticks between communities and even between municipalities.

Bearing this in mind, the objective of the present study was to detect and molecularly confirm the presence of *R. rickettsii* in *R. sanguineus* (*s.l.*) collected from free-roaming dogs in different municipalities in the south of Coahuila, where a recent outbreak of RMSF was reported.

## Methods

### Study area and samples

**Fig. 1:**
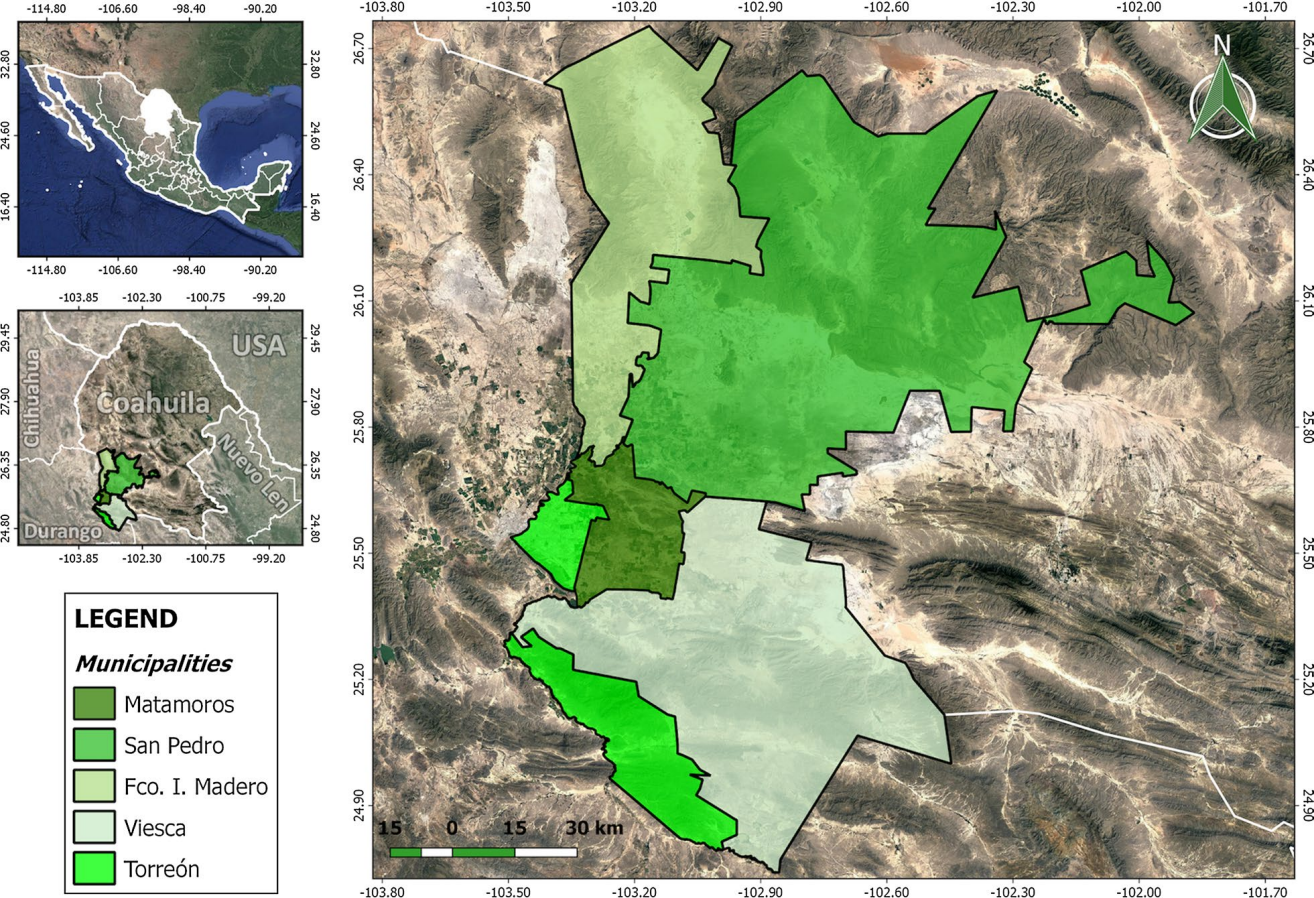
Map showing the location of the municipalities in Coahuila, Mexico, surveyed in the present study

This study was performed from September 2015 to February 2016 in 30 communities belonging to five municipalities (Torreon, San Pedro, Viesca, Francisco I. Madero and Matamoros) in the south of Coahuila (Fig. 1). This region is featured by limited water resources and a warm summer, with an annual temperature ranging between 20–22 °C but reaching around 50 °C during the hottest days of the year. The rainfall ranges between 200–250 mm per year and the average altitude is between 1000 and 1200 m above sea level. From each municipality, 60 free-roaming dogs were captured and restrained with help of people from the local communities, some of them claiming to be the dog owners, although all the dogs were captured in the streets. The sampling procedures were carried out according to the guidelines of the Federal Law for Animal Health [9]. The dogs were examined for engorged ticks, and when present, six ticks per dog were collected from most of the animals. However, the tick infestation level was very low in some cases and less than six ticks were collected. Considering that the *R. rickettsii* infection rate in ticks is very low, we focused our study on engorged ticks to increase the probability of detection, and also because a previous study showed that 100% of the engorged females became infected upon feeding on dogs with rickettsia [10]. Collected ticks were preserved in vials containing 70% ethanol and morphologically identified in the laboratory [11–13].

### DNA extraction and PCR assays

To assess the circulation of *Rickettsia* spp. in the surveyed communities, ticks collected from dogs were pooled per community (maximum six ticks per pool) (Table 1) and stored in 1.5 ml tubes (Eppendorf, Hilden, Germany) at -80 °C. Prior to DNA extraction, ticks were washed three times in distilled water for 10 min to reduce external bacteria and other possible contaminants, then dried using sterile filter paper. DNA from pooled ticks was extracted using the cetyltrimethylammonium bromide method [14].

**Table 1:** Number of ticks collected from five municipalities in the south of Coahuila, northern Mexico, according to community of origin and developmental stage. Infestation rates and mean intensity are also reported

Community	Engorged females	Engorged nymphs	Tick infestation rate (%)	Mean intensity
Congregacion Hidalgo	17	3	50	4.0
Granada	37	14	70	7.3
Vicente Guerrero	21	12	50	6.6
Solis	29	6	80	4.4
Manantial	28	10	60	6.3
El Cambio	37	2	100	3.9
La Ventana	67	6	70	10.4
San Juan de Villanueva	21	8	50	5.8
San Isidro	12	4	80	2.0
Villa de Bilbao	38	3	70	5.9
Gabino Vazquez	35	2	100	3.7
Zapata	53	2	100	5.5
El Retiro	22	2	80	3.0
Luchana	85	2	90	9.8
Concordia	20	1	80	2.6
San Miguel	19	2	100	2.1
Mayrán	20	4	100	2.4
San Nicolas	30	1	80	3.9
Lequeitio	92	15	100	10.7
Jaboncillo	27	4	80	3.9
Santo Niño	29	1	80	3.8
Hidalgo	33	16	100	4.9
El Cántabro	44	5	100	4.9
El Venado	72	10	100	8.2
La Flor de Jimulco	49	5	100	5.4
Ana	24	6	100	3.0
La Partida	23	15	100	3.8
Rancho Alegre	10	2	60	2.0
Juan Eugenio	25	6	100	3.1
La Trinidad	44	6	100	5.0
Total	1063	175	84.3	4.9

DNA samples were initially screened by a conventional PCR assay targeting the internal transcribed spacer (ITS) between the *23S* and *5S* ribosomal RNA genes (23S-5S rRNA ITS) of *Rickettsia* spp., using the primers ITS-F (5′-GAT AGG TCG GGT GTG GAA G-3′) and ITS-R (5′-TCG GGA TGG GAT CGT GTG-3′), as previously described [15]. Briefly, cycling conditions consisted of 5 min of initial denaturation at 96 °C, 35 cycles at 94 °C for 1 min, 52 °C for 1 min and 72 °C for 1 min, followed by a final extension of 5 min at 72 °C. Distilled water and a previously determined *R. rickettsii*-positive sample [16] were used as negative and positive controls, respectively.

Positive pools were further tested by a conventional PCR assay targeting the outer membrane protein A (*ompA*) gene of *Rickettsia* spp. belonging to the spotted fever group, using the primers Rr190.70p (5′-ATG GCG AAT ATT TCT CCA AAA-3′) and Rr190.701n (5′-GTT CCG TTA ATG GCA GCA TCT-3′), as described elsewhere [17, 18]. PCR thermal conditions were as follows: 3 min of initial denaturation at 95 °C, 35 cycles at 95 °C for 20 s, 46 °C for 30 s and 63 °C for 1 min, followed by a holding at 72 °C for 7 min. All amplifications were carried out in a Px2 Thermal Cycler (ThermoFisher Scientific, Waltham, MA, USA).

### DNA sequencing and phylogenetic analysis

PCR products were purified with ExoSAP-IT kit (Affymetrix, Cleveland, OH, USA), following the manufacturer’s instructions, and purified products sequenced at the Macrogen DNA Sequencing Service (Rockville, MD, USA). For phylogenetic analysis, maximum likelihood (ML) phylogenetic trees based on *Rickettsia* 23S-5S rRNA ITS and *ompA* gene sequences were inferred using Molecular Evolutionary Genetics Analysis (MEGA) software, v.6 [19]. Sequences were collected in GenBank to represent different *Rickettsia* species (using 23S-5S rRNA ITS and *ompA*) and strains (using *ompA*) as shown in results. Sequences were aligned with MAFFT (v.7) configured for the highest accuracy using the scoring matrix 200PAM/kD2, alignment strategy MAFFT-FFT-NS-I, gap opening penalty 1.53 and offset value 0.123. The best-fit model of sequence evolution was selected based on corrected Akaike information criterion and Bayesian information criterion implemented in MEGA. The Tamura 3-parameter model [20], which showed the lowest values of corrected Akaike information criterion and Bayesian information criterion (for both 23S-5S rRNA ITS and *ompA*), was chosen for the tree reconstruction. Initial trees for the heuristic search were obtained automatically in MEGA by applying neighbour-joining and BioNJ algorithms to a matrix of pairwise distances estimated using the maximum composite likelihood approach, and then selecting the topology with superior log likelihood value. Reliability of internal branches was assessed using the bootstrapping method with 1000 bootstrap replicates [21].

### Minimum infection rate

The minimum infection rate (MIR) was calculated assuming that each positive pool contained at least one positive tick, using the formula: MIR = (number of positive pools / total number of tested ticks) × 100.

## Results

Out of 300 free-roaming dogs enrolled in the present study, 253 were infested by ticks, which corresponds to an overall infestation rate of 84.3% (95% CI: 80.2–88.4%). All ticks collected were morphologically identified as *R. sanguineus* (*s.l.*). In total, 1238 specimens were collected, of which 1063 (85.9%) were engorged females and 175 (14.1%) engorged nymphs. Out of 30 pools tested, six (each six females) were found to be positive to *Rickettsia* spp. DNA (Table 2), with an MIR of 3.3% (95% CI: 1.2–7.1%).

**Table 2:** *Rickettsia* spp. identified in *Rhipicephalus sanguineus* (*sensu lato*) ticks collected from dogs from five municipalities in the south of Coahuila, northern Mexico

Municipality	Tick-infested dogs/examined dogs	Tick infestation rate (%)	Pools positive/tested to 23S-5S rRNA ITS (GenBank ID)	Pools positive/tested to *ompA* (GenBank ID)	Species identification
Francisco I. Madero	56/60	93.3	1/6 (MF925412)	1/1 (MF925420)	*R. rickettsii*
Torreon	56/60	93.3	2/6 (MF925413, MF325415)	1/2 (MF925418)	*R. rickettsii* and *R. rhipicephali*
Viesca	47/60	78.3	1/5 (MF925414)	1/1 (MF925419)	*R. rickettsii*
San Pedro	53/60	88.3	2/7 (MF925416, MF925417)	0/2	*R. rhipicephali*
Matamoros	41/60	68.3	0/6	0/0	na
Total	253/300	84.3	6/30	3/6	na

*Abbreviation*: na, not applicable

**Fig. 2:**
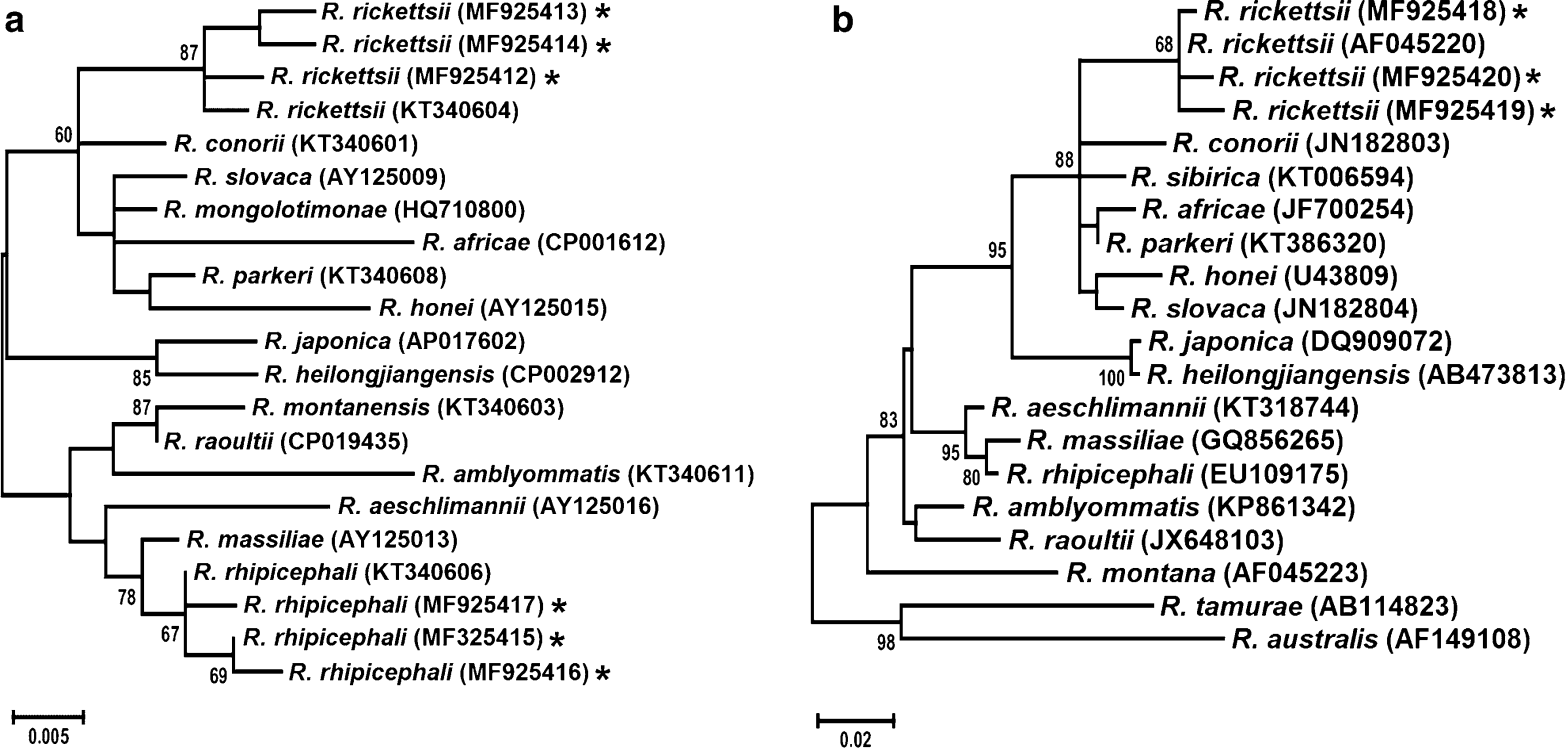
Phylogenetic analysis of *Rickettsia* spp. identified in Mexico. Maximum likelihood phylogenetic trees were inferred using 23S-5S rRNA ITS (**a**) and *ompA* sequences (**b**) of *Rickettsia* spp. identified in this study (asterisks) and other bacteria of the order Rickettsiales. The 23S-5S rRNA ITS and *ompA* sequences of Mexico clustered with *R. rickettsii* and *R. rhipicephali* reference sequences. Reliability of internal branches was assessed using the bootstrap test (1000 replicates) and only values higher than 60% are shown

By BLAST analysis, the sequences obtained from ticks collected in Francisco I. Madero, Torreon and Viesca presented 99% identity to different sequences of *R. rickettsii* from the USA (GenBank: U11022, CP003311 and CP018914). Two sequences from San Pedro and one from Torreon showed 99% identity to *Rickettsia rhipicephali* from south-eastern Brazil (GenBank: KT340606), New Mexico (USA) (GenBank: CP013133) and North Carolina (USA) (GenBank: CP003342). No positive samples were found in the municipality of Matamoros. GenBank accession numbers for the sequences generated in this study are provided in Table 2 and Fig. 2.

The phylogenetic analysis confirmed that the sequences generated in this study formed well-defined groups with other sequences from *R. rhipicephali* and *R. rickettsii*, which were supported by high bootstrap values (Fig. 2).

## Discussion

In the present study, we detected the presence of *Rickettsia* spp. DNA in *R. sanguineus* (*s.l.*) collected from free-roaming dogs in all investigated municipalities except Matamoros. *Rickettsia rickettsii* was detected in ticks from the municipalities of Francisco I. Madero, Torreon and Viesca. The absence of positive samples from Matamoros is in contrast with a previous study, which reported the presence of *R. rickettsii* DNA in *R. sanguineus* (*s.l.*) [16]. Nonetheless, this finding is not unexpected as the prevalence of infection in ticks is usually very low [1, 22]. In fact, high infection rates may be due to ticks collected from dogs with bacteraemia, as discussed elsewhere [23].

In two pools from Torreon and San Pedro, sequences with 99% of identity to *R. rhipicephali* were obtained, representing the first record of this rickettsial organism in northern Mexico. *Rickettsia rhipicephali* was originally detected in *R. sanguineus* (*s.l.*) in Mississippi, USA [24], then in several other tick species and regions of the world [25]. Because no co-infection with other species from the spotted fever group (e.g. *R. rickettsii*) has been found in ticks infected with *R. rhipicephali*, it has been speculated that this rickettsial organism of unknown pathogenicity may protect ticks from subsequent infections with pathogenic rickettsiae [25].

Although *Rickettsia* spp. DNA was detected in tick pools, the possibility that this DNA was contained in the dog’s blood ingested by the tick during feeding cannot be ruled out, particularly considering that females with varying degrees of engorgement were included in the pools. Nonetheless, it is interesting to note that the bacteraemia produced by *R. rickettsii* in dogs is transient, lasting from 3 to 13 days under experimental conditions [10, 26]. Therefore, there is also a possibility that some of these females were already infected before feeding on these dogs. As previously mentioned, *R. sanguineus* (*s.l.*) is a recognized vector of *R. rickettsii* in some epidemic foci of RMSF [27], including in northern Mexico and south-western USA [1, 3, 5], where this tick is the primary vector of this pathogen.

Although brown dog ticks are considered to display low affinity for humans [28], previous studies indicate that human parasitism may be more common than previously recognized in some regions [29–31]. Moreover, *R sanguineus* (*s.l.*) constitutes a complex of species [11, 12, 32], whose degree of affinity to humans and other non-canine hosts should be better investigated.

Even though all dogs enrolled in this study were found free roaming in the streets of the investigated municipalities, most of them are in daily contact with people. In some cases, people from the local communities declared themselves as owners of the dogs and reported that they were typically living outdoors during the day and indoors during the night. This behaviour may contribute to the introduction to ticks into the houses, potentially increasing the risk of *R. rickettsii* transmission. This highlights the need for One Health education initiatives in the RMSF-endemic regions in Mexico, to inform dog owners and the general population about responsible ownership as well as to increase awareness regarding ticks and tick control in dogs. The community-wide application of long-acting tick collars may be effective in controlling focal outbreaks [33], but should be promoted by public-private partnerships, considering that many dog owners may not be able to handle the costs of permanent tick control. Other interventions such as dog spay and neuter programs and treatment of houses against ticks may also be needed [33].

## Conclusions

The present study confirms the presence of *R. rickettsii* and *R. rhipicephali* circulating among *R. sanguineus* (*s.l.*) collected from free-roaming dogs in Coahuila, where RMSF is endemic. This suggests that free-roaming dogs may play a role in the dissemination of *R. rickettsii*-infected ticks in this region. Further research is required to assess whether ticks from free-roaming dogs could serve as indicators for the molecular xenomonitoring of *R. rickettsii* in areas witnessing epidemics of RMSF associated with massive *R. sanguineus* (*s.l.*) infestation.

## Abbreviations

RMSF: Rocky Mountain spotted fever
23S-5S rRNA ITS: internal transcribed spacer between the *23S* and *5S* ribosomal RNA genes
*ompA*: outer membrane protein A gene
MIR: minimum infection rate
*s.l.*: *sensu lato*
PCR: polymerase chain reaction
